# Does transbronchial lung cryobiopsy give useful information in asthmatic patients?

**DOI:** 10.1186/s40248-019-0176-5

**Published:** 2019-04-08

**Authors:** Sara Colella, Claudia Ravaglia, Chiara Massaccesi, Vittorio D’Emilio, Gianluca Panella, Federica Fioretti, Emanuele Giovanni Conte, Guido Collina, Riccardo Pela, Venerino Poletti

**Affiliations:** 1 Pulmonology Unit, “C. & G. Mazzoni” Hospital, Ascoli Piceno, Italy; 2 Department of Thoracic Diseases, “GB. Morgagni” Hospital, Forlì, Italy; 3 Pathology Department, “C. & G. Mazzoni” Hospital, Ascoli Piceno, Italy; 4 Department of Respiratory Diseases and Allergy, Aarhus University Hospital, Aarhus, Denmark

## Abstract

**Introduction:**

Lung biopsy in asthmatic patients is justified in case of atypical presentations of asthma, when other differential diagnoses, such as hypersensitivity pneumonitis or eosinophilic granulomatosis with polyangiitis, could be possible or for research purposes.

**Aim:**

We aim to describe the utility and the safety of TBLC (transbronchial lung cryobiopsy) in asthmatic patients, providing data on the pathological changes occurring in the airways and in the lung parenchyma.

**Methods:**

We reviewed asthmatic patients that underwent TBLC, that eventually had only a final diagnosis of asthma.

**Results:**

Three patients were detected. TBLC described pathological abnormalities in peribronchiolar and alveolar spaces already well identified with SLB (surgical lung biopsy); the pathological information provided could be useful to better understand the pathobiology of the disease. Finally, we had no complications, confirming a satisfactory safety profile of TBLC.

**Conclusion:**

We suggest the potential role of TBLC in asthmatic patients: its safety and its acceptable diagnostic accuracy lead to consider this procedure instead of SLB when histological changes in lung parenchyma are needed for the differential diagnosis. Furthermore, TLBC could be useful for research in the pathobiology of asthma and severe asthma.

## Introduction

Lung biopsy is not routinely performed in the diagnosis of asthma. However, it could be justified in case of atypical presentations of asthma, including parenchymal alterations at the CT (Computed Tomography) scan, increased FeNO (Fractional Exhaled Nitric Oxide) or increased eosinophilic count despite high doses of systemic corticosteroids, respiratory failure or DL_CO_ (Diffusion Lung capacity for Carbon Monoxide) < 70% of predicted, autoantibodies positivity, coexisting autoimmune disease, positive family history for autoimmune disease, or when other differential diagnosis, such as hypersensitivity pneumonitis or eosinophilic granulomatosis with polyangiitis [1], could be considered. Moreover, lung biopsy could be useful for research purposes, to better understand the pathobiology of asthma, especially in case of a severe disease.

Literature about this topic is sparse since invasive procedures are not out of risks in asthmatic patients and the benefits of the procedure are not always balanced with the risks.

The first reports about the pathological changes in the lung of asthmatic patients were *post-mortem* analyses and afterwards similar results were reported with SLB (surgical lung biopsy) [2] and TBB (transbronchial biopsy) [3]. It is well known that SLB has safety disadvantages over conventional TBB, despite the latter usually gives less diagnostic information than SLB. The recently introduced TBLC (transbronchial lung cryobiopsy) has the advantage to have a higher diagnostic yield than conventional TBB, [4] with a good pathological inter-observer agreement [5] but with a better safety profile than SLB; therefore, nowadays TBLC has gained ground in the diagnostic algorithm of several thoracic diseases, especially in ILDs (interstitial lung diseases) [6].

In this report, we describe the safety and the utility of TBLC in patients with an atypical presentation of asthma, providing additional evidences about the pathological picture of asthma and the safety of the procedure.

## Methods

We retrospectively reviewed our database of patients suspected for ILD with a final diagnosis of asthma that underwent cryobiopsy from September 2016 to November 2018, and we selected three patients.

Demographic, clinical, functional, radiological and pathological data were retrospectively collected.

Blood tests such as blood count, renal function, liver function, autoantibodies and serum precipitines, were obtained in every patient.

The decision of taking a biopsy was made on the abnormalities seen on the high-resolution CT-scan, along with clinical features.

TBLC procedure: cryobiopsies were performed in the Pulmonary Unit of Ascoli Piceno. Procedures were performed in general anesthesia. Patients were always intubated with a rigid tube (tracheoscope 14, Storz, Tuttlingen, Germany) and maintained in spontaneous breathing. Fogarty balloon was placed in the selected segment in order to prevent and control bleeding. Biopsies were taken either with a 1.9 mm or with a 2.4 mm probe, that was pushed forward a flexible scope until the target biopsy site was reached, under fluoroscopic guidance. Freezing time was 5 s using the larger probe and 7 s using the smaller one. In each patient 1 to 5 biopsies were taken. Each biopsy was washed out in saline and then fixed in formalin. Afterward, samples were embedded in paraffin, orientating them properly in order to maximize the area for the analysis and eventually hematoxylin- eosine slides were prepared. Specific stains (i.e.: Ziehl Neelsen, PAS) were required when deemed necessary.

For each patient, the following pathological data were recorded:

- Small airways alterations, focusing on bronchial wall abnormalities
- Description of the peribronchiolar tissue
- Abnormalities of the alveolar spaces

## Results

Demographic, clinical, functional, and radiological data are showed in Table 1.

**Table 1:** Clinical and radiological features

	1	2	3
Sex	M	F	F
Age	37	74	48
Smoking Status	No	No	No
Exposure	No	No	No
Allergies	Acarus, olive, grasses	No	No
Age onset asthma	37	74	33
FEV_1_ (% pred)	84	120	86
DL_CO_ (% pred)	74	62	68
Blood tests	Not relevant	IgE: 211	Eosinophils: 0,80 × 10^3^/mmc (11,5%) IgE: 442
Asthma therapy	Beclomethasone/Formoterol 200/6 bid	Beclomethasone/Formoterol 100/6 2 puff bid	Os: Prednisone 5 mg qd; Fluticasone/Vilanterol 184/22 qd
CT scan abnormalities	Peribronchiolar ground glass attenuation in the RUL, in the lingula and in both the posterior segments of the lower lobes. A thickening of the bronchial walls	Peribronchiolar ground glass opacities in the RUL, RLL, alveolar consolidations in the RLL and mediastinal lymphoadenopathies	Centrilobular micronodules and bronchiectasis in the medium lobe, in the right superior and inferior lobes

*RUL* right upper lobe, *RLL* right lower lobe, *LLL* left lower lobe

### Case n° 1

#### Clinical characteristics

Male, 37 years-old; he was referred for recurrent respiratory infections and cough.

No smoking history or previous toxic exposures. Allergies to acarus, olive and grasses were previously documented.

Blood tests were not relevant for any disease.

The PFTs (pulmonary function tests) showed a mild reduction of DL_CO_ and a moderate bronchial hyper-responsiveness to the methacholine challenge test.

The CT scan demonstrated peribronchiolar ground glass attenuation in the RUL (right upper lobe), in the lingula and in both the posterior segments of the lower lobes. A thickening of the bronchial walls was evident.

Bronchoalveolar lavage (BAL) showed a mixed neutrophilic and eosinophilic alveolitis. He was already under treatment with beclomethasone + formoterol 200/6 mcg, 2 puff bid.

#### Histology

*Bronchiolar wall: goblet metaplasia and eosinophilic infiltration of mucosa and submucosa. Thickening of the basal lamina.

**Fig. 1:**
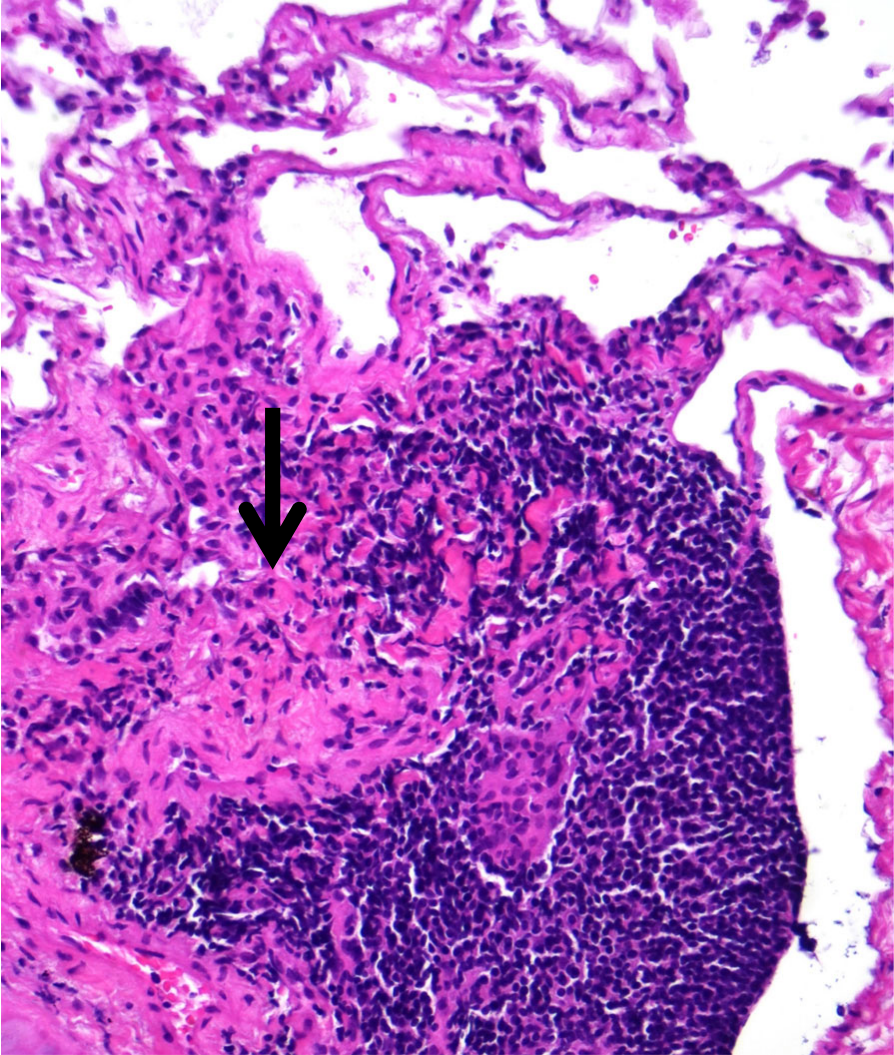
Peribronchial alveolated tissue showing a small granuloma partly sorrounded by lymphoid tissue (H&E, mid power)

*Peribronchiolar tissue: nodular lymphoid inflammation and scattered non-necrotizing granulomas (Fig. 1).

*Alveolar spaces: no inflammation.

### Case n° 2

#### Clinical characteristics

Female, 74 years-old; she was referred for a suspect of ILD found during exams done for a hyper-eosinophilic syndrome. She complained cough and mild dyspnea.

No smoking history, no previous toxic exposures nor allergies were documented.

The PFTs showed a mild obstructive pattern, reversible to bronchodilators, with hyperinflation and a moderate reduction of DL_CO_.

Increased IgE was the only abnormality detected in the blood tests.

The CT scan demonstrated peribronchiolar ground glass opacities in the RUL, RLL (right lower lobe), alveolar consolidations in the RLL and mediastinal lymphoadenopathies.

The BAL showed a lymphocytic alveolitis.

A diagnosis of bronchial asthma was eventually made, partially controlled with beclomethasone + formoterol 100/6 mcg, 2 puff bid.

#### Histology

*Bronchiolar wall: goblet metaplasia and eosinophilic infiltration of mucosa and submucosa; thickening of the basal lamina.

*Peribronchiolar tissue: nodular lymphoid inflammation with scattered eosinophils.

*Alveolar spaces: mild lymphoid interstitial inflammation with scattered eosinophils; mild macrophage accumulation in the alveolar spaces.

### Case n° 3

#### Clinical characteristics

Female, 48 years-old; she was referred for dry cough and shortness of breath. The patient did not report smoking history, nor previous toxic exposures nor allergies. Blood tests demonstrated peripheral eosinophilia and an increase of the IgE levels.

A diagnosis of intrinsic asthma was done at the age of 33 years and at the time of referral was being treated with prednisone 5 mg/qd + fluticasone/vilanterol 184/22 mcg + montelukast. She was also affected from paranasal sinusitis, treated with fluticasone furoate nasal spray.

The PFTs showed an obstructive pattern, not reversible to bronchodilator, with air trapping and a moderate reduction of DL_CO_ (FEV_1_ 86%, FEV_1_FVC 65%, RV 103%, DL_CO_ 68%- of predicted).

The CT scan demonstrated the presence of centrilobular micronodules and bronchiectasis in the medium lobe, in the right superior and inferior lobes.

The BAL showed an eosinophilic alveolitis.

#### Histology

*Bronchiolar wall: goblet metaplasia and eosinophilic and lymphocytic infiltration of mucosa and submucosa. A thickening of the basal lamina was evident. In the bronchiolar lumen accumulation of epithelial cells, eosinophils and mucous was detected.

*Peribronchiolar tissue: lymphocytic (with germinal center) and eosinophilic inflammation mainly around small vessels.

*Alveolar tissue: mild interstitial inflammation consisting of lymphocytes and scattered eosinophils.

**Fig. 2:**
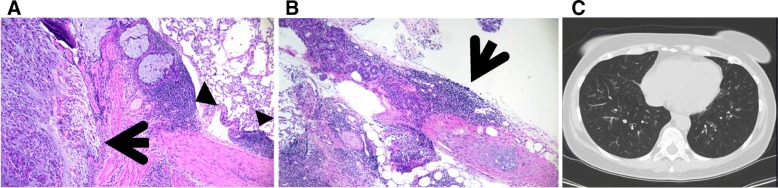
Pathological and radiological findings in a severe asthmatic patient. **a** A small bronchiole (arrow) presents goblet cell metaplasia and the lumen completely occupied by mucus, epithelial cells and eosinophils. In the peribronchial region lymphoid nodules are evident (arrowhead). **b** In the peribronchiolar alveolated tissue lymphoid infiltrates are evident (arrows) (H&E, low power). **c** High-Resolution CT scan at the lung base showing thickening of the wall and dilatation of the lumens of the bronchi and some centrilobular micronodules

Figure 2 shows the pathological and the radiological findings of case n°3.

A summary of BAL and pathological data is shown in Table 2.

**Table 2:** Summary of the BAL and pathological characteristics of each patient

Patient	BAL	Bronchiolar wall	Peribronchiolar tissue	Alveolar spaces	Vasculitis
1	Neutrophilic (72%) and eosinophilic (6%) alveolitis	Goblet metaplasia and eosinophilic infiltration of mucosa and submucosa; thickening of the basal lamina	Nodular lymphoid inflammation and scattered non-necrotizing granulomas	No inflammation	No
2	Lymphocytic (85%) alveolitis	Goblet metaplasia and eosinophilic infiltration of mucosa and submucosa; thickening of the basal lamina	Nodular lymphoid inflammation with scattered eosinophils	Mild lymphoid interstitial inflammation with scattered eosinophils; mild macrophage accumulation in the alveolar spaces	No
3	Eosinophilic (52%) alveolitis	Goblet metaplasia and eosinophilic and lymphocytic infiltration of mucosa and submucosa. A thickening of the basal lamina. In the bronchiolar lumen: accumulation of epithelial cells, eosinophils and mucous.	Lymphocytic and eosinophilic inflammation mainly around small vessels	Mild interstitial inflammation consisting of lymphocytes and scattered eosinophils	No

We did not observe any periprocedural complication, nor asthma exacerbation.

## Discussion

The knowledge of tissue abnormalities in severe asthmatic patients may provide useful information to understand the pathophysiology of the disease [7].

The histological characteristics of severe asthma [8, 9] could be characterized by small airways alterations (in bronchiolar wall and in peribronchiolar tissue), parenchymal alteration with alveolar septal mononuclear infiltrates; non-necrotizing granulomas have also been reported.

Small-airways diseases refer to both the bronchiolar wall and to the peribronchiolar tissue and include mucus plugging, epithelial detachment or epithelial metaplasia, goblet cell metaplasia or hyperplasia, bronchiolar subepithelial thickening and fibrosis, smooth muscle hypertrophy or hyperplasia and mucous gland hypertrophy [3]. Alveolar septa may be enlarged by infiltration of different inflammatory cells, most frequently mononuclear ones.

Moreover, non- necrotizing granulomas could be found and are characteristics of a different entity, called “asthmatic granulomatosis” [1, 8]. This last pathologic entity was described firstly by Wenzel et al. [10]: from a clinical point of view, patients were prevalently females, with an adult-onset disease, a family history of autoimmunity, low DL_CO_, disproportionately low FEF_25_–_75_ predicted, persistent blood eosinophils (> 200 per millimeter), FENO greater than 30 ppb despite systemic CS use and suggestion to increased responsivity to non-steroidal immunosuppressive therapy; from a pathological point of view, poorly demarcated non-necrotizing granulomas were found, along with small airways changes and marked muscular wall hypertrophy with mucus plugs.

In our case series, TBLC allowed the description of the lung parenchyma and of the distal airways very similarly to SLB. TBLC specimens, because of their dimension and the well preservation of the tissue structure, allowed to obtain similar information of SLB. We found mainly goblet cells metaplasia in the bronchial wall and infiltration of eosinophils and lymphocytes in the mucosa and in the submucosa in the bronchial wall and in the peribronchiolar tissue, as it was already described in SLB [3]. About the lung parenchyma, compared to what has been described in the literature, we found a fewer degree of inflammation in the alveolar spaces: lymphocytic aggregates in one patient, mild lymphocytic inflammation in another and no inflammation in the remaining one. In one patient, non-necrotizing granulomas were also identified. Data on the histopathology of large airways are accumulating [11]. TBLC explores mainly the lung parenchyma and in this study we hypothesize that transbronchial cryobiopsy might be useful to identify subsets of patients with peculiar asthmatic changes (that suggests connection between asthma and autoimmunity) in small airways and lung parenchyma [3]. Furthermore, we found that, as it is for ILDs, TBLC has the potential role to give the same information of SLB, without jeopardizing patient’s safety: we did not experience any complications in our patients, and this underlines the good safety profile of the procedure also in asthmatic patients [5]. Finally, we did not find any differences between the pathological picture of asthma and severe asthma; however, these results need to be confirmed in larger studies.

## Conclusion

On the basis of our experience, TLBC can be considered in asthmatic patients without additional risks, but our results need to be confirmed on a larger scale. Furthermore, the role of histology in predicting the course or how could help in the management of asthma is still not clearly defined, thus it seems reasonable to consider lung biopsy mainly when other diseases have to be ruled out; obviously, the procedure that combines less invasiveness and higher capacity to provide enough material for morphological analysis – such as TBLC- should be preferred.

## Abbreviations

BAL: Broncho Alveolar Lavage
CT: Computed Tomography
DL_CO_: Diffusion Lung capacity for Carbon Monoxide
FeNO: Fractional Exhaled Nitric Oxide
ILDs: Interstitial Lung Diseases
PFTs: Pulmonary Function Tests
RLL: Right Lower Lobe
RUL: Right Upper Lobe
SLB: Surgical Lung Biopsy
TBB: Transbronchial Biopsy
TBLC: Trans Bronchial Lung Cryobiopsy

## References

[1] Doberer D, Trejo Bittar HE, Wenzel SE (2015). Should lung biopsies be performed in patients with severe asthma?. Eur Respir Rev.

[2] Trejo Bittar HE, Doberer D, Mehrad M, Strollo DC, Leader JK, Wenzel S, et al. Histologic Findings of Severe/Therapy-Resistant Asthma from Video-assisted Thoracoscopic Surgery Biopsies. Am J Surg Pathol. 2017;41(2):182–8. 10.1097/PAS.0000000000000777 PMC5234856 28079597

[3] Balzar S, Wenzel SE, Chu HW (2002). Transbronchial biopsy as a tool to evaluate small airways in asthma. Eur Respir J.

[4] Ravaglia C, Bonifazi M, Wells AU, Tomassetti S, Gurioli C, Piciucchi S, et al. Safety and Diagnostic Yield of Transbronchial Lung Cryobiopsy in Diffuse Parenchymal Lung Diseases: A Comparative Study versus Video-Assisted Thoracoscopic Lung Biopsy and a Systematic Review of the Literature. Respiration. 2016;91(3):215–27. 10.1159/000444089 26926876

[5] Tomassetti S, Wells AU, Costabel U, Cavazza A, Colby TV, Rossi G, et al. Bronchoscopic Lung Cryobiopsy Increases Diagnostic Confidence in the Multidisciplinary Diagnosis of Idiopathic Pulmonary Fibrosis. Am J Respir Crit Care Med. 2016;193(7):745–52. 10.1164/rccm.201504-0711OC 26562389

[6] Hetzel J, Maldonado F, Ravaglia C, Wells AU, Colby TV, Tomassetti S, et al. Transbronchial Cryobiopsies for the Diagnosis of Diffuse Parenchymal Lung Diseases: Expert Statement from the Cryobiopsy Working Group on Safety and Utility and a Call for Standardization of the Procedure. Respiration. 2018;95(3):188–200. 10.1159/000484055. 10.1159/000484055 29316560

[7] Trejo Bittar HE, Yousem SA, Wenzel SE (2015). Pathobiology of severe asthma. Annu Rev Pathol..

[8] Carroll N, Elliot J, Morton A, James A (1993). The structure of large and small airways in nonfatal and fatal asthma. Am Rev. Respir Dis.

[9] Poletti V (2013). Eosinophilic bronchiolitis: is it a new syndrome?. Eur Respir J.

[10] Wenzel SE, Vitari CA, Shende M, Strollo DC, Larkin A, Yousem SA (2012). Asthmatic granulomatosis: a novel disease with asthmatic and granulomatous features. Am J Respir Crit Care Med..

[11] Menzella F, Lusuardi M, Galeone C, Facciolongo N (2017). Bronchial thermoplasty and the role of airway smooth muscle: are we on the right direction?. Ther Clin Risk Manag.

